# Systematic Review of Patient Preferences and Experiences Regarding Dietetic Outpatient Care

**DOI:** 10.1111/jhn.70056

**Published:** 2025-04-29

**Authors:** Pooja Kumar, Kelly Lambert

**Affiliations:** ^1^ School of Medical, Indigenous and Health Sciences University of Wollongong Wollongong New South Wales Australia; ^2^ Health Innovations University of Wollongong Wollongong New South Wales Australia; ^3^ Kidney Lifestyle Research Group University of Wollongong Wollongong New South Wales Australia

**Keywords:** dietitian, medical nutrition therapy, meta‐ethnography, outpatient care, patient experience, patient perception, systematic review

## Abstract

**Introduction:**

Dietitians play a crucial role in delivering medical nutrition therapy in outpatient settings where overnight admission of patients is not required. Despite the increasing focus on value‐based healthcare and patient‐reported measures (PRMs), there have been no recent reports synthesising patient experiences of dietetic outpatient care. This study aims to synthesise existing literature on patient preferences and experiences of outpatient dietetic care and to provide updated guidance for dietitians to improve patient‐centred care.

**Methods:**

A qualitative systematic review with meta‐ethnography was conducted. The review included studies that reported patient experiences of dietetic care provided at outpatient settings detailed through focus groups, interviews, surveys or questionnaires, regardless of language, year or nationality. Participants’ quotes and second‐order concepts were extracted verbatim and synthesised. Main themes and sub‐themes were then developed.

**Results:**

Five database searches yielded 5786 articles. After title and abstract screening and full‐text review, 72 articles were included. Three overarching themes were identified: (1) the process of accessing and receiving dietetic care was problematic; (2) the delivery and content of dietetic advice were suboptimal at times and (3) personal attributes of the dietitian and a desire for speciality expertise influenced perceptions of the quality of dietetic care.

**Conclusion:**

The findings from this study provide actionable insights for dietitians to tailor their outpatient services by improving accessibility, refining the delivery of care and enhancing specialised expertise to meet individual patient needs and expectations effectively.

## Summary

- This synthesis of patient preferences and experiences of outpatient dietetic care identified key areas for improvement: the process of accessing and receiving dietetic care was problematic; the delivery and content of dietetic advice were suboptimal at times and personal attributes of the dietitian and a desire for speciality expertise influenced perceptions of the quality of dietetic care.
- Activities designed to address these elements align with the principles of value‐based healthcare and may lead to improved patient satisfaction and health outcomes.

## 1 Introduction

Outpatient or ambulatory care clinics involve the provision of care in a clinical setting whereby patients are not required to be admitted or stay overnight [1, 2]. This may include clinical care provided in public or private settings and may include individual care or group attendance. Dietitians frequently provide outpatient services to deliver medical nutrition therapy and this model of care is endorsed by dietetic societies in more than 50 countries [3]. In Australia, more than 460,000 private practice appointments were reimbursed through Medicare alone in 2019 [4]. What is unclear from the literature are the voices of patients who attend these clinics and what salient features of outpatient appointments are preferable or satisfactory. A 2012 qualitative investigation of experiences of patients attending dietetic consultations for long‐term conditions (obesity, diabetes or cardiovascular disease) found that the provision of information, the approach of the dietitian and the structure of the appointment all contributed to patient satisfaction with the experience [5]. Similarly, in 2017, qualitative work on older people visiting dietetic services for malnutrition indicated the importance of regular contact, coordination and collaboration [6]. No contemporary pooled syntheses of patient experiences from a qualitative perspective have occurred. This is particularly pertinent given the widespread change in healthcare delivery following the COVID‐19 pandemic.

Other methods to measure patient experience and satisfaction with outpatient care include the use of patient‐reported measures (PRM). These measures align with global efforts to deliver value‐based healthcare and reflect the growing expectations of patients, caregivers, clinicians and the wider community [7, 8, 9]. Value‐based healthcare (VBHC) is a collaborative initiative between the government and healthcare systems to deliver care with four outcomes: (a) health outcomes that matter to patients; (b) optimal experiences of receiving care; (c) improved experiences of providing care and (d) effective and efficient care [7, 8, 9, 10, 11]. By prioritising patient outcomes above mere service volume, VBHC encourages healthcare providers to deliver personalised and patient‐centred care that meets individual expectations and needs [9].

A diverse range of PRMs are used by dietitians in the outpatient setting. Lambert et al. have identified that the wide range of measures used makes it challenging to compare patient‐reported impacts of dietetic care between speciality areas [12]. The effectiveness and efficiency of healthcare depend on managers and clinicians using evidence‐based guidelines and PRMs to drive innovation and service improvement [8, 10]. Given the importance, it is essential to synthesise the perspectives of patients engaging with dietitians in outpatient settings from both qualitative and PRM perspectives [5, 13, 14].

Therefore, the aim of this study was to (i) synthesise the existing literature regarding patient experiences and preferences in dietetic outpatient clinics and (ii) offer contemporary guidance for dietitians in clinical practice.

## 2 Materials and Methods

This qualitative systematic review synthesised data from primary studies and is reported according to the Meta‐ethnography reporting guidelines (eMERGE) and Enhancing Transparency in Reporting the Synthesis of Qualitative Research (ENTREQ) statement and Preferred Reporting Items for Systematic reviews and Meta‐Analyses (Supporting Information S1: Tables 1‐3) [15, 16, 17]. The protocol was registered on PROSPERO on 1 October 2024 (PROSPERO number CRD42024593125).

We followed the seven steps for meta‐ethnography to conduct this synthesis [18] (Supporting Information S1: Figure S1). Studies eligible for inclusion in this review were required to meet the following criteria: (a) report on dietetic care provided in outpatient settings, including private or public hospitals, clinics or other non‐admitted care environments; (b) include details on the patient experience of dietetic consultations (collected through methods such as focus groups, interviews, surveys or questionnaires) and (c) be written in any language, from any year or nationality [15, 19]. The selection criteria were developed using the PICOS approach with the following research question: Population: adults and children with a chronic condition; Intervention: medical nutrition therapy in the outpatient setting; Comparator: studies comparing usual care or with no comparator and Outcome: participants’ subjective experience of dietetic outpatient care. Five bibliographic databases were searched on the 19 of February 2024, which included Medline, PubMed, Scopus, CINAHL and Web of Science. The systematic search was strategized using Suggested Subject Terms, MeSH terms and Boolean operators to enhance search results, with search strategies shown in Supporting Information S1: Table S1. A preliminary pilot search was conducted on PubMed to evaluate the practicability of the search strategy using the search terms. Adjustment of searches was repeated using common terms used for patient‐centred care resulting in the identification of seven sentinel articles, which were retrieved [5, 6, 14, 20, 21, 22, 23]. The search terms were then adapted across databases: PubMed, Medline, Scopus and Web of Science (Supporting Information).

The Mixed Methods Appraisal Tool51 (MMAT) and Critical Appraisal Skills Programme checklist [24] were used to appraise the quality of mixed‐method and qualitative papers, respectively. Papers were assessed by two independent reviewers (P.K. and K.L.) and any disagreements about the quality appraisal were resolved via consensus. Articles retrieved from the search were imported into Covidence for screening and removal of duplicate entries. Two reviewers (P.K. and K.L.) screened the title and abstracts of all citations according to the inclusion criteria. Any disagreements during this process were resolved by discussion between the reviewers. For studies that passed the initial screening, full‐text articles were retrieved. All articles were full‐text screened by one reviewer (P.K.). A second reviewer (K.L.) examined 10% of the full‐text articles to ensure consistency in the application of inclusion criteria. In the data extraction phase, the eligible articles were imported to Dedoose Software [25] for open inductive coding. Articles were coded according to the following criteria: (a) the qualitative data and relevant second‐order constructs were directly and explicitly referring to dietetic care and (b) included free text responses from surveys and/or participant quotes from interviews. Information included in conference abstracts, abstracts and paraphrases of findings from other articles were not coded.

Data extraction focused on (a) methods used to conduct the research; (b) tools or methods used to gather patient‐reported experiences (e.g., use of PREMs) and (c) patient perspectives concerning dietetic and nutrition services [26]. Microsoft Excel was used to systematically categorise codes and concepts from eligible articles. Initial codes were developed by two researchers separately and agreed upon by consensus to support rigour and credibility within the analysis process. The codes and concepts developed were then categorised, critiqued and discussed to develop second‐order constructs (i.e., researcher interpretations of the primary data). Reciprocal translation was undertaken across studies to understand the patient experience [18, 27]. This enabled the team to interpret and collaboratively construct themes and sub‐themes [18]. These were further refined through extensive iterative discussions to generate final themes and sub‐themes. Exemplar quotes were included for each subtheme.

## 3 Results

**Figure 1:**
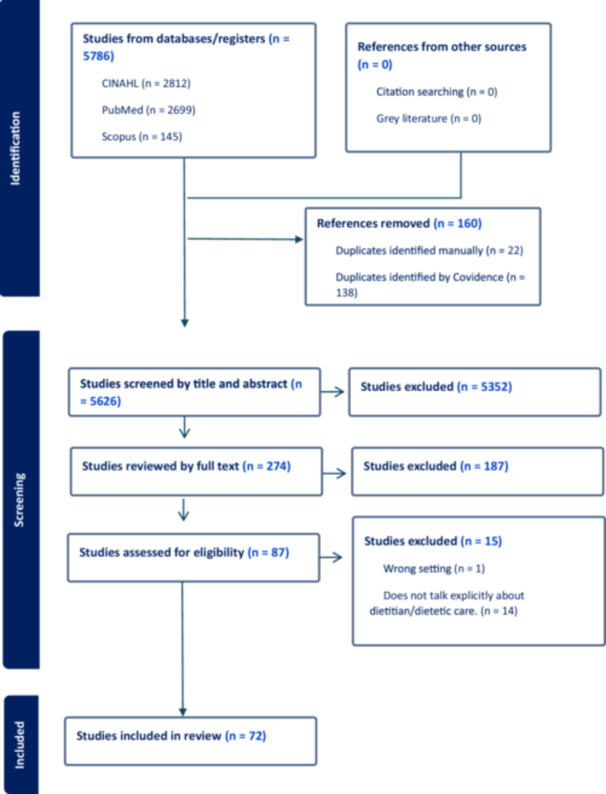
PRISMA Flowchart.

The database searches yielded 5786 articles. After 160 duplicates were removed, 5626 underwent title and abstract screening of which 274 were further assessed in full‐text review **(**Figure 1 **).** Seventy‐two articles were extracted and imported to Dedoose for coding [5, 6, 21, 28, 29, 30, 31, 32, 33, 34, 35, 36, 37, 38, 39, 40, 41, 42, 43, 44, 45, 46, 47, 48, 49, 50, 51, 52, 53, 54, 55, 56, 57, 58, 59, 60, 61, 62, 63, 64, 65, 66, 67, 68, 69, 70, 71, 72, 73, 74, 75, 76, 77, 78, 79, 80, 81, 82, 83, 84, 85, 86, 87, 88, 89, 90, 91, 92, 93, 94]. A total of 303 codes were created, and 1195 excerpts were grouped under each code.

A total of 62 qualitative papers [5, 22 28, 29, 30, 31, 32, 33, 34, 35, 21, 37, 38, 39, 40, 41, 42, 43, 44, 45, 47, 48, 49, 50, 51, 52, 53, 55, 6, 57, 58, 59, 60, 61, 62, 64, 65, 66, 68, 69, 70, 72, 73, 74, 75, 76, 77, 80, 81, 82, 83, 84, 85, 86, 88, 89, 90, 91, 92, 93, 94, 95] and 10 mixed‐method papers [36, 46, 54, 56, 63, 67, 71, 78, 79, 87] were included in the present study (Table 1). Most included studies were conducted in Australia (*n* = 40), followed by the United Kingdom (*n* = 9), the United States (*n* = 6), Canada (*n* = 5) and Ireland (*n* = 3). The publication date of papers ranged from 2001 to 2024, with 15 published after 2021 representing post‐pandemic insights. A range of qualitative methods of data collection in outpatient settings were employed in the 72 articles included in this study. Most studies utilised a single method of data collection method such as interviews (*n* = 35, 48.6%), focus groups (*n* = 6, 8.3%), surveys (*n* = 6.9%), web‐based interviews (Zoom, web‐based surveys, *n* = 4, 5.6%) and telephone interviews (*n* = 11, 15.3%). However, several studies adopted a combination of these methods (*n* = 11, 15.3%). A total of five studies reported using patient‐reported experience measures (PREMs) tool and patient‐reported outcome measure (PROMs) tools to explore patient experiences and expectations of dietetic outpatient services. Four PREMs were identified in this review, which include the Quality of Prenatal Care Questionnaire (QPCQ) [61], the CHANGE Programme Questionnaire [67], Short Assessment of Patient Satisfaction (SAPS) and Consultation and Relational Empathy (CARE) [78]. Additionally, three PROMs were also identified, which include Survivorship Clinic PROMs [81], Cachexia & Nutrition Support Service PROMs [93] and Treatment Expectations and Goals Survey Tool (TEGST) [78].

**Table 1:** Study characteristics.

Authors/year	Country/region	Study design	Outpatient setting	Data collection method	Data analysis method
Aarts and Sivapalan et al. (2017) [28]	Canada	Qualitative	Bariatric Clinic	Semi‐structured interviews	Inductive thematic
Al‐Azri and Al‐Azri et al. (2011) [29]	Muscat region, Oman	Qualitative	Primary healthcare	Semi‐structured face‐to‐face interviews	Thematic
Alberda and Alvadj‐Korenic et al. (2017) [31]	Canada	Qualitative	Primary healthcare	Semi‐structured interviews	Content
Al‐Adili and Nordgren et al. (2023) [30]	Sweden	Qualitative	Cancer clinic (Thoracic)	Semi‐structured interviews	Inductive thematic
Andersen and Linkhorst et al. (2021) [32]	Denmark	Qualitative	Primary healthcare	Semi‐structured qualitative interviews	Thematic
Arana and Valderas et al. (2016) [33]	United Kingdom	Qualitative	GP Clinic and Diabetes community centre	Focus groups	Thematic
Avgerinou and Bhanu et al. (2019) [34]	United Kingdom	Qualitative	GP clinic	One‐to‐one interviews	Inductive thematic
Baguley and Benna Doyle et al. (2024) [36]	Australia	Mixed Method	Cancer Research Centre (Prostate)	Cross‐sectional survey (Qualtrics survey) and a semi‐structured interview via Zoom.	Statistical and Thematic
Baguley and Smith‐Gillis et al. (2023) [35]	Australia	Qualitative	Cancer Research Centre (Bowel)	Semi‐structured, audio‐recorded interviews	Thematic
Ball and Davmor et al. (2016) [37]	Australia	Qualitative	Primary healthcare	Semi‐structured qualitative telephone interviews	Triangular analysis and meta‐synthesis
Ball and Davmor et al. (2016) [38]	Australia	Qualitative	Diabetes care	Semi‐structured qualitative telephone interviews	Triangular analysis and meta‐synthesis
Ball and Desbrow et al. (2014) [39]	Australia	Qualitative	Primary healthcare	Semi‐structured telephone interview	Thematic
Barnett and Campbell et al. (2020) [40]	Australia	Qualitative	Liver transplant centre	Focus groups and one‐on‐one interviews	Thematic
Beer and Lambert et al. (2024) [41]	Australia	Qualitative	Dialysis centre	Focus groups	Inductive thematic
Beirne and Andrews et al. (2023) [42]	Ireland	Qualitative	Maternal clinic	Semi‐structured focus groups	Inductive thematic
Bravo and Sabree et al. (2024) [43]	USA	Qualitative	Diabetes	Semi‐structured interviews via Zoom	Deductive thematic
Bray and Heruc et al. (2023) [44]	Australia	Qualitative	Eating disorder clinic	Semi‐structured interviews	Thematic
Burrows and Patterson et al. (2012) [21]	Australia	Qualitative	Weight loss clinic	Survey	Content
Cant (2009) [45]	Australia	Qualitative	Primary healthcare	Audio‐recorded in‐depth interviews and focus groups	Thematic
Cant (2009) [46]	Australia	Mixed Method	Primary healthcare	Semi‐structured interviews	Thematic
Chan and Lok et al. (2009) [47]	Hong Kong	Qualitative	Lifestyle Modification Programme Research	Semi‐structured interviews	Thematic
Cotugno and Ferguson et al. (2015) [48]	Australia	Qualitative	Primary healthcare	Interviews	Thematic
Dawson and Tong et al. (2020) [49]	Australia	Qualitative	Dialysis Centre	Semi‐structured interviews	Thematic
Elran‐Barak and Lewis et al. (2021) [50]	Israel	Qualitative	Eating Disorder Research	Posted messages on social media platform	Phenomenological and Content
Falbe and Friedman et al. (2017) [51]	USA	Qualitative	Primary healthcare	Semi‐structured interviews via phone	Thematic
Findlay and Rankin et al.(2020) [52]	Australia	Qualitative	Primary healthcare	Semi‐structures interviews	Inductive thematic
Foley and Houston (2014) [53]	Australia	Qualitative	Primary healthcare	Focus groups and in‐depth interviews	Thematic
Frayne and Hauck et al (2020) [54]	Australia	Mixed Method	Maternal Clinic and Mental Health	Survey	Thematic
Fry and Wilkinson et al. (2023) [55]	Australia	Qualitative	Maternal Health	Semi‐structured telephone interviews	Inductive thematic
Furness and Huggins et al. (2021) [56]	Australia	Mixed Method	Primary healthcare	Semi‐structured qualitative interviews via telephone	Deductive thematic
Gillis and Martin et al. (2018) [57]	Canada	Qualitative	ERAS programme (Colorectal)	Semi‐structured focus groups	Inductive thematic
Hancock and Bonner et al. (2012) [5]	United Kingdom	Qualitative	Primary healthcare	Interviews and focus groups	Thematic
Hazzard and Barone et al. (2017) [6]	Australia	Qualitative	Primary healthcare	Survey, face‐to‐face, semi‐structured interviews	Thematic
Hazzard and Haughton et al. (2020) [58]	Australia	Qualitative	Cancer Care	Semi‐structured interviews	Thematic
Hiatt and Young et al. (2021) [59]	Australia	Qualitative	Cancer care (Head and Neck)	Interviews	Thematic
Jager and Sande et al. (2018) [60]	Netherlands	Qualitative	Primary healthcare	Semi‐structured interviews	Deductive thematic
Jarman and Adama et al. (2018) [61]	Canada	Qualitative	Primary healthcare	Interviews and focus groups	Inductive thematic
Jones and Furlanetto et al. (2007) [62]	Scotland	Qualitative	Primary healthcare	Semi‐structured interviews	Thematic
Keaver and O'Callaghan et al. (2023) [63]	Ireland	Mixed Method	Cancer care (Breast)	Cross‐sectional survey and focus groups	Statistical and Thematic
Keaver and Richmond et al. (2022) [64]	Ireland	Qualitative	Primary healthcare	Survey	Statistical and Thematic
Kemper and Brewer et al. (2008) [65]	USA	Qualitative	Summer Camp (renal disease)	Focus group	Inductive thematic
Kitscha and Farmer et al. (2009) [66]	Alberta	Qualitative	Paediatric Weight Management	Telephone survey	Thematic
Klein and Brauer et al. (2018) [67]	Canada	Mixed Method	Primary healthcare	Survey and focus groups	Inductive thematic
Lambert and Mansfield et al. (2018) [69]	Australia	Qualitative	Research (Bariatric Surgery)	Semi‐structured interviews	Thematic
Lam and Alagoz et al. (2023) [68]	Wisconsin	Qualitative	Primary healthcare	Semi‐structured interviews	Content
Lawford and Bennell et al. (2020) [70]	Australia	Qualitative	Osteoarthritis	Semi‐structured interviews	Thematic
Loeliger and Dewar et al. (2021) [71]	Australia	Mixed Method	Primary healthcare	Survey and focus groups	Statistical and Content
MacKenzie and Grundy et al.(2014) [72]	United Kingdom	Qualitative	Allergy Community Centre	Focus groups	Statistical and Thematic
Madden and Riordan et al. (2016) [73]	United Kingdom	Qualitative	Coeliac Support Group	Telephone, face‐to‐face interview or focus group	Thematic
Mash and Cairncross (2022) [74]	South Africa	Qualitative	Primary healthcare	Focus group interviews	Framework method
McCarter and Baker et al. (2017) [77]	Australia	Qualitative	Primary healthcare	Semi‐structured telephone interviews	Thematic
Mawardi and Lestari et al. (2022) [76]	Indonesia	Qualitative	Cancer Care (Bowels)	Interviews	Phenomenological
Matsell and Sanchez‐Garcıa et al (2020) [75]	United Kingdom	Qualitative	Primary healthcare	Survey	Statistical
McMaster and Cohen et al. (2020) [78]	Australia	Mixed Method	Cancer Care (Head and Neck)	Surveys and one‐on‐one phone interviews	Statistical and Content
Morris and Herrmann et al. (2018) [22]	United Kingdom	Qualitative	Paediatric Weight Management	Semi‐structured in‐depth interviews	Phenomenological
Mutsekwa and Canavan et al. (2019) [79]	Australia	Mixed Method	Renal Disease	Survey	Thematic
Notaras and Conti et al. (2018) [80]	Australia	Qualitative	Gastroenterology Clinic	Semi‐structured interview	Inductive thematic
Obeid and Dhillon et al. (2023) [81]	Australia	Qualitative	Dialysis Centre	Semi‐structured focus groups	Framework method
Rigby and Mitchell et al. (2022) [82]	Australia	Qualitative	Cancer Care Centre	Semi‐structured telephone interviews	Content
Sharman and Hensher et al. (2015) [83]	Australia	Qualitative	Diabetes	Semi‐structured focus groups	Thematic
Siopis and Colagiuri et al. (2020) [84]	Australia	Qualitative	Primary healthcare	Semi‐structured telephone interviews	Inductive thematic
Sladdin and Chaboyer et al. (2018) [85]	Australia	Qualitative	Primary healthcare	Semi‐structured interviews	Thematic
Somerville and Burch et al. (2020) [86]	Australia	Mixed Method	Primary healthcare	Survey and semi‐structured telephone interviews	Thematic
Mari and Ball et al. (2021) [87]	Australia	Qualitative	Diabetes	Semi‐structured telephone interviews	Inductive Content
Stevenson and Tong et al. (2018) [88]	Australia	Qualitative	Dialysis Centre	Semi‐structured interviews	Thematic
Stewart and Chapple et al. (2008) [89]	United Kingdom	Qualitative	Weight management clinic	Interview	Content
Sussmann and Karen (2001) [90]	USA	Qualitative	Dialysis Centre	Semi‐structured interview	Thematic
Testa and Furness et al (2023) [91]	Australia	Qualitative	Research (Upper GI)	Telephone interviews	Thematic
Trace and Collinson et al. (2020) [92]	United Kingdom	Qualitative	Paediatric Nephrology	Telephone interviews	Inductive thematic
Vaughan and Harrison et al. (2021) [93]	Australia	Qualitative	Cancer Care	Semi‐structured interviews	Content
Warner and Tong et al. (2019) [94]	Australia	Qualitative	Primary healthcare	Semi‐structured interview	Inductive thematic
Wiley and Westbrook et al. (2013) [95]	Australia	Qualitative	Diabetes	Web‐based self‐reported survey	Thematic

The main outpatient clinical areas described were primary healthcare centres (*n* = 28, 38.9%), followed by oncology (*n* = 8, 11%), kidney disease including dialysis (*n* = 6, 8.3%), diabetes (*n* = 5, 6.9%), gastroenterology and maternal health and paediatrics (*n* = 3, 4.2% each) (Table 1). Other specific areas examined were GP and weight management clinics and other programmes such as summer camp for kidney disease or ERAS programme for colorectal cancer (*n* = 2, 2.8% each), allergy management, osteoarthritis, bariatric clinic and eating disorder management (*n* = 1, 1.4% each).

Sex distribution was reported in 53 studies (total = 2313 people), with 36.4% male participants (*n* = 843), 63.5% females (*n* = 1468) and two studies reporting 0.1% transgender individuals (*n* = 2). Of the 72 studies reviewed, only seven included children (*n* = 62) in the study population. However, the parents of these children provided explicit feedback regarding their experience of dietetic outpatient care.

### 3.1 Quality Appraisal

The CASP tool was used to appraise all qualitative papers. All 62 qualitative papers had clear aims, appropriate design and data collection methods [5, 6, 22, 29, 30, 31, 32, 33, 34, 35, 36, 21, 38, 39, 40, 41, 42, 43, 44, 46, 48, 49, 50, 51, 52, 53, 54, 56, 58, 59, 60, 61, 62, 63, 65, 66, 67, 69, 70, 71, 73, 74, 75, 76, 77, 78, 80, 82, 83, 84, 85, 86, 87, 88, 90, 91, 92, 93, 94, 95, 96]. For four papers [62, 73, 74, 81], the recruitment strategy was partially appropriate to address the aims of the research. Three papers [65, 70, 90] did not demonstrate that the relationship between the researcher and participants had been adequately considered, while 15 papers [53, 55, 60, 64, 66, 68, 72, 77, 80, 82, 83, 84, 85, 88, 95] partially demonstrated this. A detailed table is included in the Supporting Information (Supporting Information S1: Table S2).

Ten papers were appraised using the MMAT tool for mixed‐method design [36, 46, 54, 56, 63, 67, 71, 78, 79, 87] Supporting Information (Supporting Information S1: Table S3). Two papers did not demonstrate that outcome assessors were blinded to the intervention provided, raising the potential for assessment bias [56, 61].

This qualitative systematic review identified three overarching themes: (1) The process of accessing and receiving dietetic care was problematic; (2) the delivery and content of dietetic advice were suboptimal at times and (3) personal attributes of the dietitian and desire for speciality expertise influenced perceptions of the quality of dietetic care. These themes and 16 sub‐themes are shown in Table 2 with exemplar quotes.

**Table 2:** Themes and subthemes reflecting the patients’ experiences of dietetic care in outpatient settings.

Main theme	Sub‐themes	Quotes
The process of accessing and receiving dietetic care was problematic.	Availability of dietetic services	*“There are issues with access as well of course in both circumstances, but more with dietitians because you do get GPs in more areas than you do with dietitians.”* [82]
	Cost of services	“…. It had cost me quite a lot to go to the exercise classes and dietitian and…I thought what else can I do.” [83]
		“Went private. It was really frustrating. I had to pay separately to see an educator or a dietitian.” [91]
		“It would be good if it [nutrition care] were like glasses, like optometrists, it would be good if it was well covered by medical insurance” [82]
	Geographical location of dietetic services	“You know, some days it's a struggle to go to the garage and jump in the car let alone, you know, go to – go sit in the car for half an hour or 45 min to get somewhere, it's just hard to do, so to have that flexibility of being able to say, you know, well can we do it on such‐and‐such a date, yeah, makes a hell of a difference.” [52]
	Scheduling appointments and time constraints	“The time of day for clinic bookings was inconvenient, and rescheduling was a constant struggle.” [62]
	Mode of service delivery	“I think a face‐to‐face appointment is required with a dietitian, but if there was like an app or something you could pop in and out of for common questions, I think I would have liked something like that.” [59]
	Knowledge of what dietitians can offer	“The initial doctor didn't say much [about dietetic services] when I saw him.” [80]
		“Dietitians are the experts in food, and I think they're the experts in what we should be eating and what we shouldn't be eating and what to do about a healthy diet.” [35]
The delivery and content of dietetic advice were suboptimal at times.	Culturally sensitive care	“Dietary care does not always meet the expectations of migrant patients.” [57]
	Language	“Aspects of communication with Aboriginal and Torres Strait Islander patients which improve acceptability of dietetic service include showing respect towards Elders by addressing them as ‘Aunty’ or ‘Uncle’.” [49]
	Patient‐centred communication	“I went out (of the appointment) and my head was just…spinning…I was unprepared and had no idea what to expect.” [65]
	Individualised care	“No, only that she prescribed oral nutritional supplements for me, which … she wanted me to drink them and then I should eat more fat. Fat, butter, cream, the usual stuff that makes you fat. Hamburgers and pizzas and, stuff like that. That's about it.” [26]
	Coordinated care with other healthcare professionals	“They [midwives] asked me to do just like healthy living style… I put [on] a little bit of weight like during the end of the pregnancy and they said that's normal, but you can just go on the diet—they give me the booklet and I just eat few of the veggies and… healthy foods…” [51]
		“Multidisciplinary care should be at source and we shouldn't have to chase around the suburbs to find someone” [82]
	Conducive Clinic Environment	“I felt that the appointment visit was too long for my child to sit through.” [62]
Personal attributes of the dietitian and a desire for speciality expertise influenced perceptions of the quality of dietetic care.	Key attributes	“Everyone is professional, everyone is relaxed with their professionalism. So it's not scary, it's not daunting and overwhelming for the kids, which is brilliant.” [62]
		“The dietitian was polite and courteous.” [41]
		“She got to your level, she didn't judge you, she'd understand you.” [49]
	Appearance	“Oh, no! … I think it might have the effect that it was a little bit overbearing. Well, a little bit too professional, and a bit over‐reactive to what they do!” [43]
	Consistency of advice between dietitians	“I think different nutritionists have different working and presentation styles. Nutritionist A is more active in explaining the diet plan to me whereas nutritionist B is relatively passive. She will only explain more to you if you ask her” [44]
	Desire for speciality expertise	“The dietitian is experienced, she has seen many people with same disease, and she knows what she is doing.” [84]

Theme 1 **The process of accessing and receiving dietetic care was problematic.**

#### 3.1.1 Availability of Dietetic Services

Studies included in this review reported that limited availability was a significant barrier to accessing dietetic services. The issue of a scarcity of dietitians especially in remote rural areas was expressed in one study—*“It is just a matter of if people want to see one. Unless they live in a very remote rural area, I don't see any other barriers*” [84]. Concerns were expressed about the lack of dietitians with specialised knowledge able to be accessed near them—*“But I do not know a dietitian that has the background with this surgery. That's the only concern I have about being discharged”* [28]. Other issues were related to long wait times due to the shortage of dietitians in their locality—” The long waiting time. I am still waiting to see a dietitian. There is a shortage in [capital city]. I have been referred by the endocrinologist team to see a diabetes dietitian but there is a long wait. The team has told me they are flat out” [84].

#### 3.1.2 Cost of Services

The concept of financial barriers to accessing dietetic care was a recurrent subtheme, where studies highlighted cost being a burden and a reason to forgo dietetic services—“If it (dietetic care) costs any money I wouldn't go. I am retired I do not have the funds” [84]. One study equated the cost of private consultations with the quality of the session —“When I was diagnosed, I initially went to a private dietitian. The fee was very expensive. I paid $160 for the initial consult. I didn't get much out of it either” [84].

#### 3.1.3 Geographical Location of Dietetic Services

A few studies highlighted geographical challenges, including difficulties travelling long distances and cost of fuel as barriers to accessing dietetic services—*“*Well, I live a long way from the clinic, yes, I'm roughly an hour and a half away. If I had to go in every day, every week, I probably wouldn't have been able to do it. The cost of fuel would have been too much” [94]. One Australian study illustrated how the distance to dietetic clinics impacted their family and work—*“*I've had to travel from rather far away for the vast majority of it, and the girls have had to have time off school. I've had to take work off to do it. Something closer would have been much better” [78].

#### 3.1.4 Scheduling Appointments and Time Constraints

Many studies frequently mentioned the inconvenience of scheduling appointments. This was particularly challenging for those with work and family responsibilities—*“*It's just very time consuming. The clinics often run late as well and when your other children are at home being looked after by someone else, you just want to get home” [92]. Another study where parents attended clinic with their child also found the appointment to be long—“I felt that the appointment visit was too long for my child to sit through” [66]. While clinic appointments conflicted with work schedules, one participant reported difficulties with rescheduling appointments—“I had to cancel appointments due to conflicting scheduling with work hours. Rescheduling was difficult” [66].

#### 3.1.5 Mode of Service Delivery

Where face to face is considered a traditional method, telehealth has become a positive alternative method of service delivery after the COVID pandemic—“Yeh telehealth, particularly during Covid, now find it very useful” [41]. Participants in many studies also preferred telehealth to maintain continuity of care—“……the option to continue consultations with dietitians, in person or via phone, was available and perceived helpful” [31]. Others highlighted group sessions to be more beneficial—“In a group setting it is easier to connect with other people that have the same condition and get motivated” [84].

#### 3.1.6 Knowledge of What Dietitians Can Offer

Significant gaps were identified in public knowledge regarding referral processes and understanding of the role of dietitians and their services—*“*The only way I found out about the [nutrition service] was that there was a flyer in the oncologist's rooms. So, the onus was on me… Who knows how I would have found that kind of help” [35]. Studies frequently expressed confusion about dietitians’ scope of expertise in comparison to fellow healthcare professionals such as nutritionists—“I would go to a nutritionist I imagine rather than a dietitian. I don't actually know really what a dietitian does” [39]. Furthermore, the advice of GPs was trusted over that of dietitians—*“*I trust my doctor's advice more than anyone else. They [GPs] know more about you in your entirety, so can suggest stuff that is actually manageable for you. A nutrition specialist doesn't have any relationship with you and they might suggest things that wouldn't work” [39]. This lack of clear understanding of the dietetic role and lack of previous exposure to dietitians led to limited use of dietetic services and unfulfilled expectations.

Theme 2 **The delivery and content of dietetic advice were suboptimal at times.**

#### 3.1.7 Culturally Sensitive Care

A specific concern expressed among many studies was cultural sensitivity, describing encounters with dietitians, which on occasions did not align with their cultural dietary habits—“It is important to have dietitians that understand different cultures. E.g. I am Chinese. You cannot recommend to Chinese people to eat European food. It is easier to follow a diet that is closer to your culture” [84] and “I don't think they know much about Fijian food here” [88].

#### 3.1.8 Language

The language used by dietitians influences patient experience. For example, one study described how the service experience was perceived as impersonal and felt like being disciplined—“It's very impersonal and I felt like I was a naughty girl with the principal, and I wasn't actually going to get any answers and was told I had done bad” [78]. Another study highlighted “disliked the use of jargon” [6].

#### 3.1.9 Patient‐Centred Communication

The communication style of the dietitian played an important role in patient satisfaction, where participants desired patient‐centred communication—“The reality is every person is going to be different, and ah, I think the fact is that you talk one on one, like with [my coach] he can actually ah judge what your condition is and um change the program to um to actually what's happening to you rather than a generalization” [94]. Other participants described that the approach of delving deeper into concepts without judgement was preferred “… Going more in depth with concepts for people with higher health literacy…” [55] and “She got to your level, she didn't judge you, she'd understand you” [53].

#### 3.1.10 Individualised Care

The importance of individualised care was highlighted by many studies and many participants expressed their dissatisfaction with generic dietary advice that did not account for their personal health needs, preferences or cultural needs. For example, this approach led to seeking alternative sources of dietary advice: *“*The guidelines are not suitable to what I need. I used Facebook and the internet. It is about carbs… They [dietitians]put you on all this education and they don't give you good advice. Now I am doing keto and I am sure it will help. Most of the people I heard of are doing it and said it is the best” [84]. In contrast, dietitian demeanour including being friendly and understanding their child's situation was highly valued—“It's just their friendliness and their warmness and their understanding of [my son] you know and the circumstances. Everybody's an individual and I like that. And I understand they deal with other people as well” [78]. Other studies highlighted a strong preference to see the same dietitian every time they attended dietitian clinic—“Coming back (to the same dietitian after transplant) was good…I liked to speak to (the dietitian)…who knew me already” [69].

#### 3.1.11 Coordination of Care With Other Healthcare Professionals

The coordination of dietetic care with other healthcare professionals was highly valued. Care coordination led to a perception that dietitians were more fully informed and knowledgeable if there was a clear connection between the dietitian and other members of the care team—*“*Yeah, she (metabolic doctor) helped me stick to my diet because she was pretty much my main source of information. See, they've worked with a lot of metabolic disorders and stuff, I mean, all of them over here (at the speciality centre), so they know what they're talking about” [65] and “Going to the doctor is the first stop for many people, and a GP is far more qualified and have had a lot more training on physiology and the human body than other health professionals” [38]. One study identified that the care was further enhanced with interprofessional coordination between the dietitian and other healthcare professionals—“I felt having access to a Registered Dietitian complimented the care I received from my family doctor, OB/GYN well” [61].

#### 3.1.12 Conducive Clinic Environment

The physical environment and clinic space were identified as a key factor influencing patient experience. Participants in one study, particularly those with children, described the clinic to be unwelcoming and unaccommodating for their children—“The clinic environment provided no entertainment for my children. It was not kid‐friendly” [66]. In the same study, another participant expressed that the clinic space was too small and often not conducive to a positive experience—“Overall esthetics of the clinic room: “I felt that the clinic appointment was in too small a room” [66].

Theme 3 **Personal attributes of the dietitian and a desire for speciality expertise influenced perceptions of the quality of dietetic care.**

#### 3.1.13 Key Attributes

Professionalism and the demeanour of dietitians influenced the patient experience of dietetic consults. Many studies described dietitians as professional, courteous and empathetic who made the sessions a pleasant experience—*“*I respect her professionality [sic] …” [5], “I entered the session with great fear. The dietitian looked at me and tried to understand why I was shivering. She gave me a glass of water. She closed the opened window. She offered me a hot beverage […] Slowly, I was able to talk again” [50] and “(Dietitian) is lovely, which made it a much more pleasant appointment” [79]. However, one participant perceived dietitians as intimidating, which negatively impacted the participant's willingness to continuity of care—“I don't need to go in to have them affirm for me what a failure I am[tearfully]… I don't want to see that dietitian again. There's no way I would go now [tearfully]” [68].

#### 3.1.14 Appearance

Patient experience was also influenced by the appearance of dietitians. Business suits were perceived to be intimidating and overly formal impacting patient's willingness to engage —*“*Oh, if I walked in and the dietitian was like that (dressed in business suit) I think I would feel very intimidated! … Well, she'd be looking down at me!” [46]. Another participant in the same study equated a dietitian physical appearance with high‐quality practice—“She was a young, healthy looking sort of person; Fit! She looked like she practiced what she preached” [46].

#### 3.1.15 Consistency of Advice Between Dietitians

Numerous studies reported problems with inconsistency in dietary advice between dietitians—“When I was in Taiwan I saw two different dietitians, I got two different opinions. When I see one in Adelaide, it's different again” [88] and “…Then I moved into a different suburb and my doctor referred me to a different dietitian. She gave me a different plan but there was no variety. She told me to increase the protein in my diet. Since I saw her, I have put on weight. It is not good” [88]. Inconsistency between dietetic advice and written dietary materials was also noted—“In earlier stages, they [a dietitian] said to me watermelon is out, now in the last book I got from Diabetes SA, it says they can't wait for summer because we can all splurge into the watermelon… so I'm getting a bit confused” [38].

#### 3.1.16 Desire for Speciality Expertise

Specialised expertise was strongly desired across studies and perceived to enhance the care they would receive from dietitians. When unable to access specialised care, it was noted that there is a need for dietitians and other specialists to undertake further training to meet their specific health needs—“I do not want to lose them [the dietitians] as a resource. My family physician can probably refer me, or I can find a dietitian anywhere. But I do not know a dietitian that has the background with this surgery. That's the only concern I have about being discharged” [28] and “Perhaps more training is needed on this front with diabetic specialist nurses and dietitians, they need to be given more instruction from their mentors or whoever as to what they should and should not be telling people” [33].

## 4 Discussion

This study provides a comprehensive contemporary synthesis of patient experiences and preferences in dietetic outpatient clinics. The findings revealed critical barriers to accessing and receiving dietetic care, suboptimal delivery of care and the influence of personal and professional attributes of dietitians on patient experience. Patient preferences of dietetic care in this review may guide systematic changes to improve accessibility and delivery of care of dietetic services aligned to patient needs.

Barriers to accessing dietetic services were numerous and are a potential area for improvement. Barriers included the scarcity of dietitians, long waiting times, high costs and geographic limitations and were perceived to exacerbate health disparities. These barriers reflect broader systemic issues within healthcare that disproportionately affect the vulnerable population, particularly those in regional and remote areas and those with limited financial support [84, 87]. To address this, health systems need to consider the distribution of dietitians in underserved regions. Other suggestions to address these barriers include suggestions to expand dietetic services in underserved areas. This could potentially utilise innovative service delivery models such as telehealth, which have played a pivotal role in healthcare during the COVID‐19 pandemic [41]. Other suggestions include the use of mobile applications to access dietitians and services. This may be particularly for those who face geographical, family and work balance and mobility challenges [31, 56, 63, 66, 84, 94, 96]. Other suggestions include offering flexible appointment times, which include evening and weekend options to accommodate the work and carer responsibilities of individuals, hence improving continuity of care [33, 78].

The high cost of dietetic care was identified as a significant barrier and may be associated with the underutilisation of dietetic services [84, 87, 95]. Suggestions to address this included expanding medical insurance coverage, providing government subsidies for dietetic services and providing incentives such as parking vouchers [66, 86]. To actualise this, professional dietetic bodies should undertake advocacy efforts with healthcare policymakers and the government. Efforts to reform and implement policies to reduce financial barriers would encourage the utilisation of these services and may improve health outcomes [7, 8, 10, 13].

A substantial gap in public awareness and understanding of dietitians’ role and what they have to offer was also apparent in this review. Participants compared and confused dietitians with nutritionists and utilised other health professionals such as general practitioners to provide nutritional advice over that of dietitians [39, 52, 84]. The medical profession were also identified as gatekeepers to referrals to dietetic services. Improved advocacy about the role and capacity of dietitians and the benefits of dietetic care are needed [86]. This approach may prevent patients from utilising social media for nutritional advice [84]. Interestingly, dietitians may wish to capitalise on findings that when participants achieved success with dietary interventions they felt motivated to engage other family members and those in their immediate social network to see a dietitian [67, 84].

The experience of dietetic care varied according to cultural sensitivity, the language used to deliver content, person‐centred communication skills, individualisation of advice, collaboration with other health professionals and the physical clinic environment. Many participants identified that dietitians did not always meet the cultural needs of patients [60, 84, 88] and the use of medical jargon confused participants [6]. Dietitians therefore must be attentive to and should not underestimate the importance of providing culturally safe care. Knowledge of diverse cultural beliefs around food, health and eating are required in addition to culturally appropriate education resources [97]. The acceptability of dietetic services also depends on the relationships and trust developed with some cultural groups [53].

The communication style of dietitians plays a crucial role in patient adherence to dietary recommendations and enhancing continuity of care. A person‐centred and individualised approach by dietitians made patients feel understood and created a sense of accountability [41]. A desire for meal plans and other actionable information was also expressed frequently, and a particular desire for a flexible approach to eating and not one size fits all [55, 69, 78, 94]. The volume of information often overwhelmed patients, as identified in across various studies [41, 52, 69, 81] in this review. Although dietitians were perceived as indispensable and motivating, patients expressed a preference for group‐sessions. Many parents attending dietitian clinics for their children's health shared their nutrition journey with others, which provided additional motivation and support [78]. Participants generally desired longer consultations with more intense education at the initial stages of dietetic intervention [5, 37, 47, 84], which is also essential for building rapport and trust and fostering a positive therapeutic relationship. Current evidence from one study by Chan et al. [47] suggested dietetic consultation duration generally approximates between 20 and 40 min in Australia and the United Kingdom. This is clearly inadequate for new patients and not sufficient according to most patients in this study.

While dietary advice was often received from other health professionals, such as GPs and midwives, many participants questioned why dietitians were not part of the multidisciplinary team [5]. Collaboration and interprofessional coordination between dietitians and other healthcare professionals have been shown to enhance patient outcomes and satisfaction [5, 44, 51, 6, 93]. The physical environment of the clinic also influences the overall patient experience. Participants in this review expressed disappointment with clinic environments not being family‐friendly [66]. Suggestions to improve the outpatient clinic space included ensuring adequate room in the waiting area and providing child‐friendly amenities, both of which contribute to a more welcoming and comfortable environment [66].

The final aspect that was identified to influence the patient experience of outpatient dietetic services was the personal and professional attributes of the dietitian. These attributes included professionalism, politeness and non‐judgmental and empathetic approaches, which made the appointments pleasant [5, 21, 50, 53, 66, 68, 79]. Participants desired dietitians to be cheerleaders who showed the patient the ‘rules of the game’ and guided them to success. Dietitian appearance was also discussed with attention to attire, leading to perceptions of credibility and professionalism. Highly professional business attire was seen as intimidating by participants and untidiness was associated with disrespect [46]. There was no clear suggestion for a particular type of attire for dietitians; however, under‐dressing was also identified by participants to be inappropriate, which may adversely affect communication [46].

Dietitians were generally considered experts in nutrition information and described as role models for healthy living [46]. However, inconsistency in advice between dietitians was identified as an important concern by numerous participants in this review. For example, discrepancies in advice often occur at a local/institutional level between clinical specialties [37, 73, 88]. Inconsistent advice was perceived to be confusing [80, 88] and impacted trust in dietetic services. Some participants also reported scepticism that dietitians could truly understand or empathise with the lived experience of chronic conditions [86].

Speciality expertise in dietetic care is a professional attribute that was highly valued by participants in this review. Participants desired receival of nutrition care from dietitians with specialised expertise in clinical areas of importance to them. However, the scarcity of specialist dietitians or the lack of specialised knowledge of dietitians was frequently encountered [28, 33, 88]. Patients also strongly coveted receiving dietetic care from the same dietitian as they were aware of a patient's history and background [69, 73, 84].

A detailed list of recommendations is included to help optimise patient experiences in outpatient clinics (Table 3). These recommendations have been categorised into key areas: the process of advice and the content of advice and dietitian attributes. We suggest they will assist dietitians in advocating for patient needs, enhance clinic and practices in a manner that is aligned to meet patient needs and increase patient satisfaction and improve overall health outcomes.

**Table 3:** Recommendations for clinical practice to optimise patient experiences of outpatient clinics.

Advice delivery framework	Factors influencing patient care	Recommendations
Process of advice	Flexible Appointments	Consider parking availability, travel time and ways to reduce the burden on patients. Provide flexibility in appointment delivery, including appointment lengths and scheduling outside business hours to suit families and caregivers.
	Clinic Services	Providing complimentary parking vouchers or clear directions on how to arrive at the venue to improve access to services. Expand clinic services by offering early morning or late afternoon appointments to accommodate diverse schedules. Offer follow‐up appointments using flexible delivery options where relevant.
	Public awareness of dietitian role	GPs and other healthcare professionals should share success stories with prospective patients. Promotional material about the role of the dietitian in various speciality areas beyond weight loss.
	Capturing of experiences	Consider the inclusion of three patient‐reported measures: a generic health‐related quality‐of‐life tool, an experience measure and at least 1 clinical or direct nutrition‐related measure. This will enable dietitians to fully capture the impact of their care on patients and conduct economic evaluations if necessary.
Content of advice	Mode of delivery	Offer virtual consultations that include practical assessments, such as reviewing the contents of a patient's fridge and offering personalised suggestions. Use multiple communication methods (e.g., text, email) and provide timely reminders to enhance personalised service and build trust with patients. Offer multiple modes of delivery, such as telehealth, group sessions, tours and talks to keep people engaged and meet practical advice needs
	Clinic environment	Ensure the clinic environment is child‐friendly or family‐oriented when relevant. Provide entertainment for children and create a comfortable, inviting atmosphere in clinics
	Family Involvement	Involve families in food‐related decisions and offer family‐focused approaches, such as creating healthy eating plans that the entire family can follow.
	Patient Motivation	Sharing ‘insider knowledge’ i.e. stories of other patients successfully achieving similar goals Encourage prospective patients to seek dietetic services
	Individualised and Personalised Care	Avoid a one‐size‐fits‐all approach and take time to offer individualised care, adapting recommendations based on each patient's unique needs. Engaging patients in personal discussions helps them develop their own ideas Diet sheets should be tailored with specific recommendations on what to eat and avoid. Focus on individualised care rather than general assumptions, taking time to understand each patient's unique circumstances and adapting solutions accordingly. Consider follow‐up with individual appointments following group attendance.
	Indigenous Perspectives	Consistency with the same dietitian is important for building trust in Indigenous communities, along with a strong local presence and knowledge of traditional foods.
	Visual Information on Food	Provide more visual information on food options available at supermarkets, potentially through educational materials or tours.
	Regular updates	Provide patients with regular updates, such as monthly feedback on blood test results, via SMS or phone to encourage timely behaviour changes.
Dietitian attributes	Respect and Courtesy	Treat patients with courtesy and respect, showing care through small gestures like offering a cup of tea or greeting them warmly to encourage engagement.
	Appearance	Ensure dietitians maintain a professional appearance appropriate to the clientele and setting, as this can impact patients’ trust and their perception of the care they receive.

One key strength of this study is that it is a comprehensive synthesis of patient‐reported experiences and preferences regarding dietetic outpatient care. The meta‐ethnography approach generated novel insights into a phenomenon that exceeds the limitations of any one study. This work also expands on the findings of a previous systematic review, which synthesised encounters with dietitians across various healthcare settings—including acute hospital experiences—as well as perspectives from other stakeholders [14]. This meta‐synthesis thereby enabled a deeper exploration of the outpatient experience and the generation of specific recommendations for dietetic care. However, this study also had limitations. No reference or citation screening was conducted, which may have missed other relevant studies. This review was not able to describe or compare the dietetic models of care practised in each country of the included studies. This limits the ability to fully understand the context of the patient experience and thus generalisability of some recommendations. Future research should explore the experiences of specific populations, such as children, culturally diverse groups and transgender individuals, as these groups have been underrepresented in existing studies. More research is also required in regions such as Oceania, Africa, Europe and Asia as many of the challenges could potentially be common in developing countries, which increases health disparity among the population. Additionally, policies regarding access to dietetic care may be warranted and understanding the perspectives of all stakeholders involved in policy change would be informative.

## 5 Conclusion

This systematic review synthesised patient experiences and preferences of dietetic care provided at outpatient settings. Several critical challenges were identified, which can be addressed systematically to improve the delivery of dietetic care. The recommendations discussed in this study align with the principles of value‐based healthcare, which if implemented would enhance the accessibility, affordability and effective delivery of dietetic services, leading to improved patient satisfaction and health outcomes. Despite several challenges identified, positive aspects of dietetic care such as professionalism, personal attributes and psychosocial support should be upheld to improve the therapeutic alliance between dietitians and their patients.

## Author Contributions

**Pooja Kumar, Kelly Lambert:** project conceptualisation, data collection, data analysis, drafting final version. **Pooja Kumar:** writing the initial draft.

## Conflicts of Interest

The authors declare no conflicts of interest.

## Supporting information

suppmaterialsv2.

## Data Availability

The data that support the findings of this study are available from the corresponding author upon reasonable request.
