# Supplementary material for: Systematic Review of Patient Preferences and Experiences Regarding Dietetic Outpatient Care
Source: J Hum Nutr Diet. 2025 Apr 29;38(3):e70056. doi: 10.1111/jhn.70056 (PMC12038784; doi:10.1111/jhn.70056)
Supplement: Supplementary file 1 — suppmaterialsv2. [file JHN-38-0-s001.docx]

**Supplementary Materials**

**Supplementary Figure S1: Steps in meta-ethnographic synthesis**

Diagram adapted from Toey et al (2014) BMC Med Res Method, 14:80 and Sattar et al (2021) BMC Health Serv Res (21:50)

**Supplementary Table S1: Search Strategy**

|  | **Search Strategy** | **Number of Articles Retrieved** |
| --- | --- | --- |
|  | **PubMed** | |
| # 1 | *ambulatory care facilities OR outpatient service OR health services need and demands OR outpatients OR health service accessibility OR outpatient clinics OR ambulatory care OR access to primary care OR primary health care OR primary care setting* | 1,034,277 |
| # 2 | *Nutrition Services OR Nutritional Counsel* OR nutrition consult OR nutrition counsel* OR dietitian referral OR dietician services OR Nutritional Support Team* | 89,159 |
| # 3 | *patient satisfaction OR patient feelings OR patient experiences OR patient perception OR patient attitudes OR patient views OR patient opinions OR patient feelings* | 1,261,945 |
| **# 4** | **#1 AND #2 AND #3** | **2,699** |
|  | **Scopus** | |
| # 1 | *Outpatient Service OR ambulatory care facilities OR outpatients OR outpatient clinics OR ambulatory care OR access to primary care OR primary health care OR primary care setting* | 576,281 |
| # 2 | *Nutrition Services OR Nutritional Counsel* OR nutrition consult OR nutrition counsel OR dietitian referral OR dietician services OR Nutritional Support Team* | 390 |
| # 3 | *Patient Satisfaction OR Patient Attitude OR patient experiences OR patient perception OR patient views OR patient opinions OR patient feelings* | 36,812 |
| **# 4** | **#1 AND #2 AND #3** | **145** |
|  | **Medline** | |
| # 1 | *Outpatient Service OR ambulatory care facilities OR outpatients OR outpatient clinics OR ambulatory care OR access to primary care OR primary health care OR primary care setting* | 183,817 |
| # 2 | *Nutrition Services OR Nutritional Counsel* OR nutrition consult OR nutrition counsel OR dietitian referral OR dietician services OR Nutritional Support Team* | 14,201 |
| # 3 | *Patient Satisfaction OR Patient Attitude OR patient experiences OR patient perception OR patient views OR patient opinions OR patient feelings* | 93,539 |
| **# 4** | **#1 AND #2 AND #3** | **123** |
|  | **CINAHL** | |
| # 1 | *Outpatient Service + OR ambulatory care facilities OR outpatients OR outpatient clinics OR ambulatory care OR access to primary care OR primary health care OR primary care setting* | 34,734 |
| # 2 | *Nutrition Services + OR Nutritional Counseling + OR nutrition consult OR nutrition counsel OR dietitian referral OR dietician services OR Nutritional Support Team* | 6,671 |
| # 3 | *Patient Satisfaction + OR Patient Attitude OR patient experiences OR patient perception OR patient views OR patient opinions OR patient feelings* | 196,306 |
| **# 4** | **#1 AND #2 AND #3** | **2812** |
|  | **Web of Science** | |
| # 1 | *Outpatient Service OR ambulatory care facilities OR outpatients OR outpatient clinics OR ambulatory care OR access to primary care OR primary health care OR primary care setting* | 115,310 |
| # 2 | *Nutrition Services OR Nutritional Counsel* OR nutrition consult OR nutrition counsel OR dietitian referral OR dietician services OR Nutritional Support Team* | 2,880 |
| # 3 | *Patient Satisfaction OR Patient Attitude OR patient experiences OR patient perception OR patient views OR patient opinions OR patient feelings* | 142,351 |
| **# 4** | **#1 AND #2 AND #3** | **7** |

("ambulatory care facilities"[MeSH Terms] OR ("ambulatory"[All Fields] AND "care"[All Fields] AND "facilities"[All Fields]) OR "ambulatory care facilities"[All Fields] OR ("ambulatory care"[MeSH Terms] OR ("ambulatory"[All Fields] AND "care"[All Fields]) OR "ambulatory care"[All Fields] OR ("outpatient"[All Fields] AND "service"[All Fields]) OR "outpatient service"[All Fields]) OR (("health services needs and demand"[MeSH Terms] OR ("health"[All Fields] AND "services"[All Fields] AND "needs"[All Fields] AND "demand"[All Fields]) OR "health services needs and demand"[All Fields] OR ("health"[All Fields] AND "services"[All Fields] AND "needs"[All Fields]) OR "health services needs"[All Fields]) AND ("demand"[All Fields] OR "demanded"[All Fields] OR "demanding"[All Fields] OR "demands"[All Fields])) OR ("outpatient s"[All Fields] OR "outpatients"[MeSH Terms] OR "outpatients"[All Fields] OR "outpatient"[All Fields]) OR (("health services"[MeSH Terms] OR ("health"[All Fields] AND "services"[All Fields]) OR "health services"[All Fields] OR ("health"[All Fields] AND "service"[All Fields]) OR "health service"[All Fields]) AND ("access"[All Fields] OR "accessed"[All Fields] OR "accesses"[All Fields] OR "accessibilities"[All Fields] OR "accessibility"[All Fields] OR "accessible"[All Fields] OR "accessing"[All Fields])) OR ("ambulatory care facilities"[MeSH Terms] OR ("ambulatory"[All Fields] AND "care"[All Fields] AND "facilities"[All Fields]) OR "ambulatory care facilities"[All Fields] OR ("outpatient"[All Fields] AND "clinics"[All Fields]) OR "outpatient clinics"[All Fields]) OR ("ambulatory care"[MeSH Terms] OR ("ambulatory"[All Fields] AND "care"[All Fields]) OR "ambulatory care"[All Fields]) OR ("access to primary care"[MeSH Terms] OR ("access"[All Fields] AND "primary"[All Fields] AND "care"[All Fields]) OR "access to primary care"[All Fields]) OR ("primary health care"[MeSH Terms] OR ("primary"[All Fields] AND "health"[All Fields] AND "care"[All Fields]) OR "primary health care"[All Fields]) OR (("primary health care"[MeSH Terms] OR ("primary"[All Fields] AND "health"[All Fields] AND "care"[All Fields]) OR "primary health care"[All Fields] OR ("primary"[All Fields] AND "care"[All Fields]) OR "primary care"[All Fields]) AND ("setting"[All Fields] OR "setting s"[All Fields] OR "settings"[All Fields]))) AND ((("nutrition s"[All Fields] OR "nutritional status"[MeSH Terms] OR ("nutritional"[All Fields] AND "status"[All Fields]) OR "nutritional status"[All Fields] OR "nutrition"[All Fields] OR "nutritional sciences"[MeSH Terms] OR ("nutritional"[All Fields] AND "sciences"[All Fields]) OR "nutritional sciences"[All Fields] OR "nutritional"[All Fields] OR "nutritional"[All Fields] OR "nutrition"[All Fields] OR "nutritive"[All Fields]) AND ("service"[All Fields] OR "service s"[All Fields] OR "serviced"[All Fields] OR "services"[All Fields] OR "services s"[All Fields] OR "servicing"[All Fields])) OR (("nutrition s"[All Fields] OR "nutritional status"[MeSH Terms] OR ("nutritional"[All Fields] AND "status"[All Fields]) OR "nutritional status"[All Fields] OR "nutrition"[All Fields] OR "nutritional sciences"[MeSH Terms] OR ("nutritional"[All Fields] AND "sciences"[All Fields]) OR "nutritional sciences"[All Fields] OR "nutritional"[All Fields] OR "nutritional"[All Fields] OR "nutrition"[All Fields] OR "nutritive"[All Fields]) AND "counsel*"[All Fields]) OR (("nutrition s"[All Fields] OR "nutritional status"[MeSH Terms] OR ("nutritional"[All Fields] AND "status"[All Fields]) OR "nutritional status"[All Fields] OR "nutrition"[All Fields] OR "nutritional sciences"[MeSH Terms] OR ("nutritional"[All Fields] AND "sciences"[All Fields]) OR "nutritional sciences"[All Fields] OR "nutritional"[All Fields] OR "nutritional"[All Fields] OR "nutrition"[All Fields] OR "nutritive"[All Fields]) AND ("consultancies"[All Fields] OR "consultancy"[All Fields] OR "consultant s"[All Fields] OR "consultants"[MeSH Terms] OR "consultants"[All Fields] OR "consultant"[All Fields] OR "consultative"[All Fields] OR "consulter"[All Fields] OR "consulters"[All Fields] OR "referral and consultation"[MeSH Terms] OR ("referral"[All Fields] AND "consultation"[All Fields]) OR "referral and consultation"[All Fields] OR "consult"[All Fields] OR "consultation"[All Fields] OR "consultations"[All Fields] OR "consulted"[All Fields] OR "consulting"[All Fields] OR "consults"[All Fields])) OR (("nutrition s"[All Fields] OR "nutritional status"[MeSH Terms] OR ("nutritional"[All Fields] AND "status"[All Fields]) OR "nutritional status"[All Fields] OR "nutrition"[All Fields] OR "nutritional sciences"[MeSH Terms] OR ("nutritional"[All Fields] AND "sciences"[All Fields]) OR "nutritional sciences"[All Fields] OR "nutritional"[All Fields] OR "nutritional"[All Fields] OR "nutrition"[All Fields] OR "nutritive"[All Fields]) AND "counsel*"[All Fields]) OR (("dietitian s"[All Fields] OR "nutritionists"[MeSH Terms] OR "nutritionists"[All Fields] OR "dietitian"[All Fields] OR "dietitians"[All Fields]) AND ("referral and consultation"[MeSH Terms] OR ("referral"[All Fields] AND "consultation"[All Fields]) OR "referral and consultation"[All Fields] OR "referral"[All Fields] OR "referrals"[All Fields] OR "referrer"[All Fields] OR "referrers"[All Fields])) OR (("nutritionists"[MeSH Terms] OR "nutritionists"[All Fields] OR "dietician"[All Fields] OR "dieticians"[All Fields]) AND ("service"[All Fields] OR "service s"[All Fields] OR "serviced"[All Fields] OR "services"[All Fields] OR "services s"[All Fields] OR "servicing"[All Fields])) OR (("nutritional support"[MeSH Terms] OR ("nutritional"[All Fields] AND "support"[All Fields]) OR "nutritional support"[All Fields]) AND "Team"[All Fields])) AND ("patient satisfaction"[MeSH Terms] OR ("patient"[All Fields] AND "satisfaction"[All Fields]) OR "patient satisfaction"[All Fields] OR (("patient s"[All Fields] OR "patients"[MeSH Terms] OR "patients"[All Fields] OR "patient"[All Fields] OR "patients s"[All Fields]) AND ("emotions"[MeSH Terms] OR "emotions"[All Fields] OR "feeling"[All Fields] OR "feelings"[All Fields] OR "feels"[All Fields])) OR (("patient s"[All Fields] OR "patients"[MeSH Terms] OR "patients"[All Fields] OR "patient"[All Fields] OR "patients s"[All Fields]) AND ("experience"[All Fields] OR "experience s"[All Fields] OR "experiences"[All Fields])) OR (("patient s"[All Fields] OR "patients"[MeSH Terms] OR "patients"[All Fields] OR "patient"[All Fields] OR "patients s"[All Fields]) AND ("percept"[All Fields] OR "perceptibility"[All Fields] OR "perceptible"[All Fields] OR "perception"[MeSH Terms] OR "perception"[All Fields] OR "perceptions"[All Fields] OR "perceptional"[All Fields] OR "perceptive"[All Fields] OR "perceptiveness"[All Fields] OR "percepts"[All Fields])) OR (("patient s"[All Fields] OR "patients"[MeSH Terms] OR "patients"[All Fields] OR "patient"[All Fields] OR "patients s"[All Fields]) AND ("attitude"[MeSH Terms] OR "attitude"[All Fields] OR "attitudes"[All Fields] OR "attitude s"[All Fields])) OR (("patient s"[All Fields] OR "patients"[MeSH Terms] OR "patients"[All Fields] OR "patient"[All Fields] OR "patients s"[All Fields]) AND ("viewed"[All Fields] OR "viewing"[All Fields] OR "viewings"[All Fields] OR "views"[All Fields])) OR (("patient s"[All Fields] OR "patients"[MeSH Terms] OR "patients"[All Fields] OR "patient"[All Fields] OR "patients s"[All Fields]) AND ("attitude"[MeSH Terms] OR "attitude"[All Fields] OR "opinion"[All Fields] OR "opinions"[All Fields] OR "opinion s"[All Fields] OR "opinionated"[All Fields]

**Supplementary Table S2.** Qualitative papers evaluated using the CASP Tool

| **Author/Year** | **Q1** | **Q2** | **Q3** | **Q4** | **Q5** | **Q6** | **Q7** | **Q8** | **Q9** | **Q10** |
| --- | --- | --- | --- | --- | --- | --- | --- | --- | --- | --- |
| Aarts et al (2017) | Yes | Yes | Yes | Yes | Yes | Yes | Yes | Yes | Yes | Valuable |
| Al-Azri et al (2011) | Yes | Yes | Yes | Yes | Yes | Yes | Yes | Yes | Yes | Valuable |
| Alberda et al (2017) | Yes | Yes | Yes | Yes | Yes | Yes | Yes | Yes | Yes | Valuable |
| Al‐Adili et al (2023) | Yes | Yes | Yes | Yes | Yes | Yes | Yes | Yes | Yes | Valuable |
| Andersen et al (2021) | Yes | Yes | Yes | Yes | Yes | Yes | Yes | Yes | Yes | Valuable |
| Arana et al (2016) | Yes | Yes | Yes | Yes | Yes | Yes | Yes | Yes | Yes | Valuable |
| Avgerinou et al (2019) | Yes | Yes | Yes | Yes | Yes | Yes | Yes | Yes | Yes | Valuable |
| Baguley et al (2023) | Yes | Yes | Yes | Yes | Yes | Yes | Yes | Yes | Yes | Valuable |
| Ball et al (2016) | Yes | Yes | Yes | Yes | Yes | Yes | Yes | Yes | Yes | Valuable |
| Ball et al (2016) | Yes | Yes | Yes | Yes | Yes | Yes | Yes | Yes | Yes | Valuable |
| Ball et al (2014) | Yes | Yes | Yes | Yes | Yes | Yes | Yes | Yes | Yes | Valuable |
| Barnett et al (2020) | Yes | Yes | Yes | Yes | Yes | Yes | Yes | Yes | Yes | Valuable |
| Beer et al (2024) | Yes | Yes | Yes | Yes | Yes | Yes | Yes | Yes | Yes | Valuable |
| Beirne et al (2023) | Yes | Yes | Yes | Yes | Yes | Yes | Yes | Yes | Yes | Valuable |
| Bravo et al (2024) | Yes | Yes | Yes | Yes | Yes | Yes | Yes | Yes | Yes | Valuable |
| Bray et al (2023) | Yes | Yes | Yes | Yes | Yes | Yes | Yes | Yes | Yes | Valuable |
| Burrowsa et al (2012) | Yes | Yes | Yes | Yes | Yes | Yes | Yes | Yes | Yes | Valuable |
| Cant (2009) | Yes | Yes | Yes | Yes | Yes | Yes | Yes | Yes | Yes | Valuable |
| Chan et al (2009) | Yes | Yes | Yes | Yes | Yes | Yes | Yes | Yes | Yes | Valuable |
| Cotugno et al (2015) | Yes | Yes | Yes | Yes | Yes | Yes | Yes | Yes | Yes | Valuable |
| Dawson et al (2020) | Yes | Yes | Yes | Yes | Yes | Yes | Yes | Yes | Yes | Valuable |
| Elran-Barak et al (2021) | Yes | Yes | Yes | Yes | Yes | Yes | Yes | Yes | Yes | Valuable |
| Falbe et al (2017) | Yes | Yes | Yes | Yes | Yes | Yes | Yes | Yes | Yes | Valuable |
| Findlay et al (2020) | Yes | Yes | Yes | Yes | Yes | Yes | Yes | Yes | Yes | Valuable |
| Foley and Houston (2014) | Yes | Yes | Yes | Yes | Partially | Yes | Yes | Yes | Yes | Valuable |
| Fry et al (2023) | Yes | Yes | Yes | Yes | Partially | Yes | Yes | Yes | Yes | Valuable |
| Gillis et al (2018) | Yes | Yes | Yes | Yes | Yes | Yes | Yes | Yes | Yes | Valuable |
| Hancock et al (2012) | Yes | Yes | Yes | Yes | Yes | Yes | Yes | Yes | Yes | Valuable |
| Hazzard et al (2017) | Yes | Yes | Yes | Yes | Yes | Yes | Yes | Yes | Yes | Valuable |
| Hazzard et al (2020) | Yes | Yes | Yes | Yes | Yes | Yes | Yes | Yes | Yes | Valuable |
| Hiatt et al (2021) | Yes | Yes | Yes | Yes | Yes | Yes | Yes | Yes | Yes | Valuable |
| Jager et al (2018) | Yes | Yes | Yes | Yes | Partially | Yes | Yes | Yes | Yes | Valuable |
| Jones et al (2007) | Yes | Yes | Yes | Partially | Yes | Yes | Yes | Yes | Yes | Valuable |
| Keaver et al (2022) | Yes | Yes | Yes | Yes | Partially | Yes | Yes | Yes | Yes | Valuable |
| Kemper et al (2008) | Yes | Yes | Yes | Yes | No | Yes | Yes | Yes | Yes | Valuable |
| Kitscha et al (2009) | Yes | Yes | Yes | Yes | Partially | Yes | Yes | Yes | Yes | Valuable |
| Lambert et al (2018) | Yes | Yes | Yes | Yes | Yes | Yes | Yes | Yes | Yes | Valuable |
| Lam et al (2023) | Yes | Yes | Yes | Yes | Partially | Yes | Yes | Yes | Yes | Valuable |
| Lawford et al (2020) | Yes | Yes | Yes | Yes | No | Yes | Yes | Yes | Yes | Valuable |
| MacKenzie et al (2014) | Yes | Yes | Yes | Yes | Partially | Yes | Yes | Yes | Yes | Valuable |
| Madden et al (2016) | Yes | Yes | Yes | Partially | Yes | Yes | Yes | Yes | Yes | Valuable |
| Mash and Cairncross (2022) | Yes | Yes | Yes | Partially | Yes | Yes | Yes | Yes | Yes | Valuable |
| McCarter et al (2017) | Yes | Yes | Yes | Yes | Partially | Yes | Yes | Yes | Yes | Valuable |
| Mawardi et al (2022) | Yes | Yes | Yes | Yes | Yes | Yes | Yes | Yes | Yes | Valuable |
| Matsell et al (2020) | Yes | Yes | Yes | Yes | Yes | Yes | Yes | Yes | Yes | Valuable |
| Morris et al (2018) | Yes | Yes | Yes | Yes | Yes | Yes | Yes | Yes | Yes | Valuable |
| Notaras and Conti et al (2018) | Yes | Yes | Yes | Yes | Partially | Yes | Yes | Yes | Yes | Valuable |
| Obeid et al (2023) | Yes | Yes | Yes | Partially | Yes | Yes | Yes | Yes | Yes | Valuable |
| Rigby et al (2022) | Yes | Yes | Yes | Yes | Partially | Yes | Yes | Yes | Yes | Valuable |
| Sharman et al (2015) | Yes | Yes | Yes | Yes | Partially | Yes | Yes | Yes | Yes | Valuable |
| Siopis et al (2020) | Yes | Yes | Yes | Yes | Partially | Yes | Yes | Yes | Yes | Valuable |
| Sladdin et al (2018) | Yes | Yes | Yes | Yes | Partially | Yes | Yes | Yes | Yes | Valuable |
| Mari et al (2021) | Yes | Yes | Yes | Yes | Yes | Yes | Yes | Yes | Yes | Valuable |
| Stevenson et al (2018) | Yes | Yes | Yes | Yes | Partially | Yes | Yes | Yes | Yes | Valuable |
| Stewart et al (2008) | Yes | Yes | Yes | Yes | Yes | Yes | Yes | Yes | Yes | Valuable |
| Sussmann and Karen (2001) | Yes | Yes | Yes | Yes | No | Yes | Yes | Yes | Yes | Valuable |
| Testa et al (2023) | Yes | Yes | Yes | Yes | Yes | Yes | Yes | Yes | Yes | Valuable |
| Trace et al (2020) | Yes | Yes | Yes | Yes | Yes | Yes | Yes | Yes | Yes | Valuable |
| Vaughan et al (2021) | Yes | Yes | Yes | Yes | Yes | Yes | Yes | Yes | Yes | Valuable |
| Warner et al (2019) | Yes | Yes | Yes | Yes | Yes | Yes | Yes | Yes | Yes | Valuable |
| Wiley et al (2013) | Yes | Yes | Yes | Yes | Partially | Yes | Yes | Yes | Yes | Valuable |

**Supplementary Table S3.** Quality Appraisal of Mixed Method Papers using MMAT Tool

| **Category of study designs** | **Methodological quality criteria questions** | Baguley et al (2024) | Cant (2009) | Frayne et al (2020) | Furness et al (2021) | Jarman et al (2018) | Keaver et al (2023) | Klein et al (2018) | Loeliger et al (2021) | McMaster et al (2020) | Mutsekwa et al (2019) | Somerville et al (2020) |
| --- | --- | --- | --- | --- | --- | --- | --- | --- | --- | --- | --- | --- |
| Screening questions | **S1.** | Yes | Yes | Yes | Yes | Yes | Yes | Yes | Yes | Yes | Yes | Yes |
|  | **S2.** | Yes | Yes | Yes | Yes | Yes | Yes | Yes | Yes | Yes | Yes | Yes |
| 1. Qualitative | **1.1.** | Yes | Yes | Yes | Yes | Yes | Yes | Yes | Yes | Yes | Yes | Yes |
|  | **1.2.** | Yes | Yes | Yes | Yes | Yes | Yes | Yes | Yes | Yes | Yes | Yes |
|  | **1.3.** | Yes | Yes | Yes | Yes | Yes | Yes | Yes | Yes | Yes | Yes | Yes |
|  | **1.4.** | Yes | Yes | Yes | Yes | Yes | Yes | Yes | Yes | Yes | Yes | Yes |
|  | **1.5.** | Yes | Yes | Yes | Yes | Yes | Yes | Yes | Yes | Yes | Yes | Yes |
| 2. Quantitative randomised controlled  trials | **2.1.** | Yes | Yes | Yes | Yes | Yes | Yes | Yes | N/A | N/A | N/A | N/A |
|  | **2.2.** | Yes | Yes | Yes | Yes | Yes | Yes | Yes | N/A | N/A | N/A | N/A |
|  | **2.3.** | Yes | Yes | Yes | Yes | Yes | Yes | Yes | N/A | N/A | Yes | N/A |
|  | **2.4.** | Yes | Yes | Yes | Can't tell | No | Yes | Yes | N/A | N/A | N/A | N/A |
|  | **2.5.** | Yes | Yes | Yes | Yes | Yes | Yes | Yes | N/A | N/A | Yes | N/A |
| 5. Mixed methods | **5.1.** | Yes | Yes | Yes | Yes | Yes | Yes | Yes | Yes | Yes | Yes | Yes |
|  | **5.2.** | Yes | Yes | Yes | Yes | Yes | Yes | Yes | Yes | Yes | Yes | Yes |
|  | **5.3.** | Yes | Yes | Yes | Yes | Yes | Yes | Yes | Yes | Yes | Yes | Yes |
|  | **5.4.** | Yes | Yes | Yes | Yes | Yes | Yes | Yes | Yes | Yes | Yes | Yes |
|  | **5.5.** | Yes | Yes | Yes | Yes | Yes | Yes | Yes | Yes | Yes | Yes | Yes |
